# The effect of 5 alpha-reductase inhibitor therapy on prostate cancer detection in the era of multi-parametric magnetic resonance imaging

**DOI:** 10.1038/s41598-019-54464-9

**Published:** 2019-11-28

**Authors:** Jung Kwon Kim, Hak Jong Lee, Sung Il Hwang, Gheeyoung Choe, Hak Ju Kim, Sung Kyu Hong

**Affiliations:** 1 Department of Urology, Seoul National University Bundang Hospital, Seongnam, Korea; 2 Department of Radiology, Seoul National University Bundang Hospital, Seongnam, Korea; 3 Department of Pathology, Seoul National University Bundang Hospital, Seongnam, Korea; 4 Department of Urology, Seoul National University College of Medicine, Seoul, Korea

**Keywords:** Surgical oncology, Prostate

## Abstract

We aimed to evaluate the effect of 5 alpha-reductase inhibitor (5-ARI) treatment on prostate cancer (PCa) and clinically significant PCa (csPCa) detection in patients undergoing transrectal ultrasound (TRUS)/magnetic resonance imaging (MRI) fusion biopsy in the current era of multi-parametric MRI (mpMRI). We retrospectively reviewed our TRUS/MRI fusion biopsy database (n = 706). Eighty (11.3%) patients who had used 5-ARI for more than one year at the time of biopsy were stratified as 5-ARI group. Subsequently, we performed comparative analyses of 5-ARI and non-5-ARI groups. csPCa was defined by a Gleason score ≥3 + 4 in a single biopsy core. Chi-squared test was used to evaluate the performance of mpMRI in predicting PCa/csPCa between the two groups. Multivariate logistic regression analyses were performed to evaluate the significant variables associated with PCa detection. There were no significant differences in PCa/csPCa detection rates between 5-ARI and non-5-ARI groups (all, P > 0.05). In multivariate logistic regression analyses for the evaluation of variables associated with csPCa detection, age (odds ratio [OR], 1.062; 95% confidence interval [CI], 1.035–1.090; P < 0.001), pre-biopsy PSA (OR, 1.062; 95% CI, 1.034–1.090; P < 0.001), prostate volume on TRUS (OR, 0.956; 95% CI, 0.943–0.970, P < 0.001), and PI-RADsV2 category (OR, 5.528; 95% CI, 3.017–10.131; P < 0.001) were found to be significant predictors. However, 5-ARI had no significant association with PCa detection (P = 0.384). Conclusively, 5-ARI therapy did not adversely affect PCa/csPCa detection after TRUS/MRI fusion biopsy, which suggests that exposure to 5-ARI may not impair the performance of mpMRI.

## Introduction

In recent years, two landmark studies (PROMIS [Prostate MR imaging study] and PRECISION [Prostate Evaluation for Clinically Important Disease: Sampling Using Image Guidance or Not?] trials) have led to the emergence of the era of multiparametric magnetic resonance imaging (mpMRI) in the detection and management of prostate cancer (PCa)^1,2^. The latest European Association of Urology (EAU) guidelines also strongly recommend mpMRI both (1) biopsy-naïve men and (2) before repeat biopsy when clinical suspicion of PCa persists despite previous negative biopsies^3^.

5-alpha-reductase inhibitors (5-ARIs) have been widely used for the treatment of benign prostate hyperplasia (BPH)^4^. 5-ARIs are known to be associated with a 25% reduction in the overall prostate volume after 3 to 6 months of exposure^5^. Previous studies have investigated the expanded clinical use of 5-ARIs in PCa^6–9^. In a large randomized study of 6729 patients (REDUCE [Reduction by Dutasteride of Prostate Cancer Events] trial), Andriole *et al*.^6^ reported that 5-ARI (dutasteride) reduced the period prevalence of PCa by 24% compared with placebo. However, the mechanism of 5-ARI in PCa prevalence reduction is still elusive.

Currently, with the wide use of mpMRI, several studies have investigated the effect of 5-ARIs on mpMRI in PCa patients^10–13^. In a randomized, double-blind, placebo-controlled trial involving a total of 42 patients (MAPPED [MRI in Primary Prostate cancer after Exposure to Dutasteride] trial), Moore *et al*.^10^ reported that dutasteride was associated with a significant (48%) reduction in PCa volume in T2-weighted imaging (T2WI) compared with placebo (P < 0.0001). However, other studies investigating the changes in quantitative parameters of PCa and noncancerous lesions have shown conflicting results^11–13^. Giganti *et al*.^11^ found that dutasteride administered 0.5 mg daily for 6 months did not significantly influence the T2WI values in men under active surveillance for PCa. Conversely, in a retrospective study of 20 patients, Starobinets *et al*.^13^ demonstrated that PCa discrimination was lower with T2WI, but was higher based on functional MR measures in a 5‐ARI group compared with controls.

Thus, the aim of the present study was to evaluate the effect of 5-ARI on PCa and clinically significant PCa (csPCa) detection in patients who underwent transrectal ultrasound (TRUS)/MRI fusion biopsy in clinical practice. We also evaluated the pathological differences between 5-ARI and non-5-ARI groups.

## Materials and Methods

### Ethics statement

The Institutional Review Board of Seoul National University Bundang Hospital approved this study (Approval number: B-1706/402-115). A written informed consent of patients was waived by the Institutional Review Board as this was a retrospective study. Personal identifiers were completely deleted such that data were analyzed anonymously. Our study was conducted according to the ethical standards of the 1964 Declaration of Helsinki and its later amendments.

### Study cohort

From September 2015, two high-volume radiologists (S.I.H and H.J.L) in our institution conducted the TRUS/MRI fusion biopsy. We retrospectively reviewed our institutional TRUS/MRI fusion biopsy database between September 2015 and March 2018. Finally, a total of 706 patients were included.

### mpMRI protocol and image interpretation

All mpMRIs were performed using a 3-T system (Achieva Tx and Ingenia; Philips, the Netherlands) with a phase-array cardiac 6-channel coil without the endorectal coil. The mpMRI comprised axial T2-weighted imaging (T2WI), T1/T2-weighted registered imaging (T1/T2RI), diffusion-weighted imaging (DWI) with corresponding apparent-diffusion coefficient (ADC) maps and dynamic contrast enhancement (DCE). Detailed protocols were described in our previous reports^14,15^. All images were reviewed by two high-volume radiologists (H.J.L. and S.I.H.) who had more than 20 years of experience in interpreting prostate MRI using a Picture Archiving and Communication Systems workstation (PACS, INFINITT Technology, Seoul, Korea). All lesions were graded by the level of suspicion for csPCa based on ADC maps and T2WI using Prostate Imaging Reporting and Data System version 2 (PI-RADSv2) from 1 to 5, as follows^16^: grade 1, highly unlikely; grade 2, unlikely; grade 3, equivocal; grade 4, likely; and grade 5, highly likely. Up to two index lesions were chosen per patient.

### TRUS/MRI fusion biopsy protocol

The 3-T mpMRI was performed before biopsy in all patients. All images obtained before early 2016 were re-reviewed according to PI-RADSv2. The fusion imaging technique (Volume Navigation; GE Healthcare, USA) with an electromagnetic field tracking system was used. Before the study, the axial MR images were uploaded from the PACS archive to the TRUS machine. Later, the registration between the TRUS and MR images was performed to fuse both images accurately. In case of two index lesions in the same patient, the registrations were performed again for subsequent lesions after the first biopsy. All TRUS-guided biopsies were performed with a Logiq E9 US machine (GE Healthcare, USA) equipped with a 5–9 MHz multi-frequency endocavitary probe by the same uroradiologist who had conducted the image fusion. An 18-gauge, 20-cm automatic cutting needle and an automated biopsy gun (ACECUT, TSK Laboratory, Japan) were used.

Index lesions of PIRADSv2 category ≥3 were classified as MRI-positive; in contrast, PIRADSv2 category ≤2 groups were considered as MRI-negative. Two cores of additional biopsy were performed for each index lesion under TRUS/MRI fusion. Accordingly, a maximum of four additional biopsies were obtained per patient followed by 12 cores of randomized systematic biopsy. In the MRI-negative group, two cores of additional biopsy were conducted in the transition zone, followed by systematic biopsy. csPCa was defined by the presence of a Gleason score (GS) ≥3 + 4 in a single biopsy core^2^.

### Radical prostatectomy protocol

A subsequent radical prostatectomy (RP) after diagnosis of localized or locally advanced PCa was conducted by several surgeons (S.E.L., S.S.B., S.L., and S.K.H.) using a robotic system (da Vinci Surgical System, Intuitive Surgical Inc., Sunnyvale, CA, USA) or open retropubic approach. All pathological specimens were evaluated by a staff pathologist (G.C.) who had genitourinary expertise.

### Statistical analyses

The following variables were compared between 5-ARI and non-5-ARI groups: age, body mass index (BMI), pre-biopsy prostate-specific antigen (PSA) level, doubled pre-biopsy PSA level in 5-ARI group^6^, biopsy and pathologic GS, prostate volume on TRUS, PSA density (PSAD, serum PSA level/prostate volume)^17^, and PI-RADSv2 category.

Comparative analyses of the two groups were conducted using Chi-squared test for categorical variables, and independent t-test or Mann-Whitney U test for continuous variables. Chi-squared test was also used to evaluate the differences in performance of mpMRI in predicting PCa/csPCa between the two groups. Multivariate logistic regression analyses were performed to evaluate significant variables associated with PCa/csPCa detection. Subsequently, we also performed subgroup analysis according to the PI-RADSv2 categorical groups. In the subgroup of patients who underwent subsequent RP after TRUS/MRI fusion biopsy in our institution, Chi-squared test was used to compare pathologic outcomes including GS upgrading between 5-ARI and non-5-ARI groups. All statistical analyses were performed using IBM SPSS Statistics ver. 22.0 (Armonk, NY, USA), statistical package for R, ver. 2.13.2 (R Foundation for Statistical Computing [http://www.r-project.org/]). Statistical significance was considered when the two-sided p value was less than 0.05.

## Results

### Baseline characteristics

A total of 80 (11.3%) patients, who were treated with 5-ARI for more than one year at the time of TRUS/MRI fusion biopsy, were stratified as 5-ARI group. Baseline characteristics of 5-ARI and non-5-ARI groups are summarized in Table 1. There was significant difference between the two groups in age (median [interquartile range], 69.0 [63.0–73.0] vs. 65.0 [58.0–71.0], respectively, P < 0.001). However, we found no significant differences among the other variables including BMI, pre-biopsy PSA, doubled pre-biopsy PSA level in 5-ARI group^6^, prostate volume on TRUS, PSAD, PI-RADSv2 category, and number of biopsy cores. Regarding the duration of medication, 35 (43.7%) patients were exposed to long-term (≥3 years) 5-ARI therapy.

**Table 1:** Baseline characteristics of 5-ARI and non-5-ARI groups in the total cohort.

N (%) or Median (IQR)	Non-5-ARI group (N = 626)	5-ARI group (N = 80)	P
Age	65.0 (58.0–71.0)	69.0 (63.0–73.0)	<0.001
BMI	24.5 (22.9–26.1)	25.0 (22.6–26.5)	0.664
Pre-biopsy PSA	7.23 (4.91–11.05)	5.56 (3.92–11.50)	0.734
Pre-biopsy PSA (doubling)	7.23 (4.91–11.05)	11.12 (7.84–22.99)	0.546
Prostate volume, total	38.7 (28.5–51.1)	41.0 (27.0–57.0)	0.278
PSA density	0.18 (0.12–0.31)	0.13 (0.09–0.32)	0.084
PI-RADSv2 category			0.083
≤2	151 (24.2%)	22 (27.5%)	
3	216 (34.5%)	30 (37.5%)	
≥4	259 (41.4%)	28 (35.1%)	
Number of biopsy cores (Mean ± SD)	14.2 ± 0.7	14.3 ± 0.8	0.492
Pathologic outcomes			
Total group	N = 626	N = 80	
All cancer	228 (36.4%)	29 (36.3%)	0.976
Clinically significant cancer*	152 (24.3%)	23 (28.8%)	0.169
MRI-positive group**	N = 475 (75.9%)	N = 58 (72.5%)	0.493
All cancer	184 (38.7%)	23 (39.7%)	0.888
Clinically significant cancer*	137 (28.8%)	21 (36.2%)	0.074
Duration of medication (years)			
Median (IQR)	—	3.0 (2.0–5.0)	
1–3, N (%)	—	45 (56.3%)	
≥3, N (%)	—	35 (43.7%)	
≥5, N (%)	—	20 (25.0%)	

5-ARI, 5 alpha-reductase inhibitor; BMI, body mass index; IQR, interquartile range; PI-RADSv2, Prostate Imaging–Reporting and Data System version 2; PSA, prostate-specific antigen; SD, standard deviation.

*GS ≥ 3 + 4.

**PI-RADSv2 category ≥ 3, who received targeted biopsy.

### TRUS/MRI fusion biopsy outcomes

**Figure 1:**
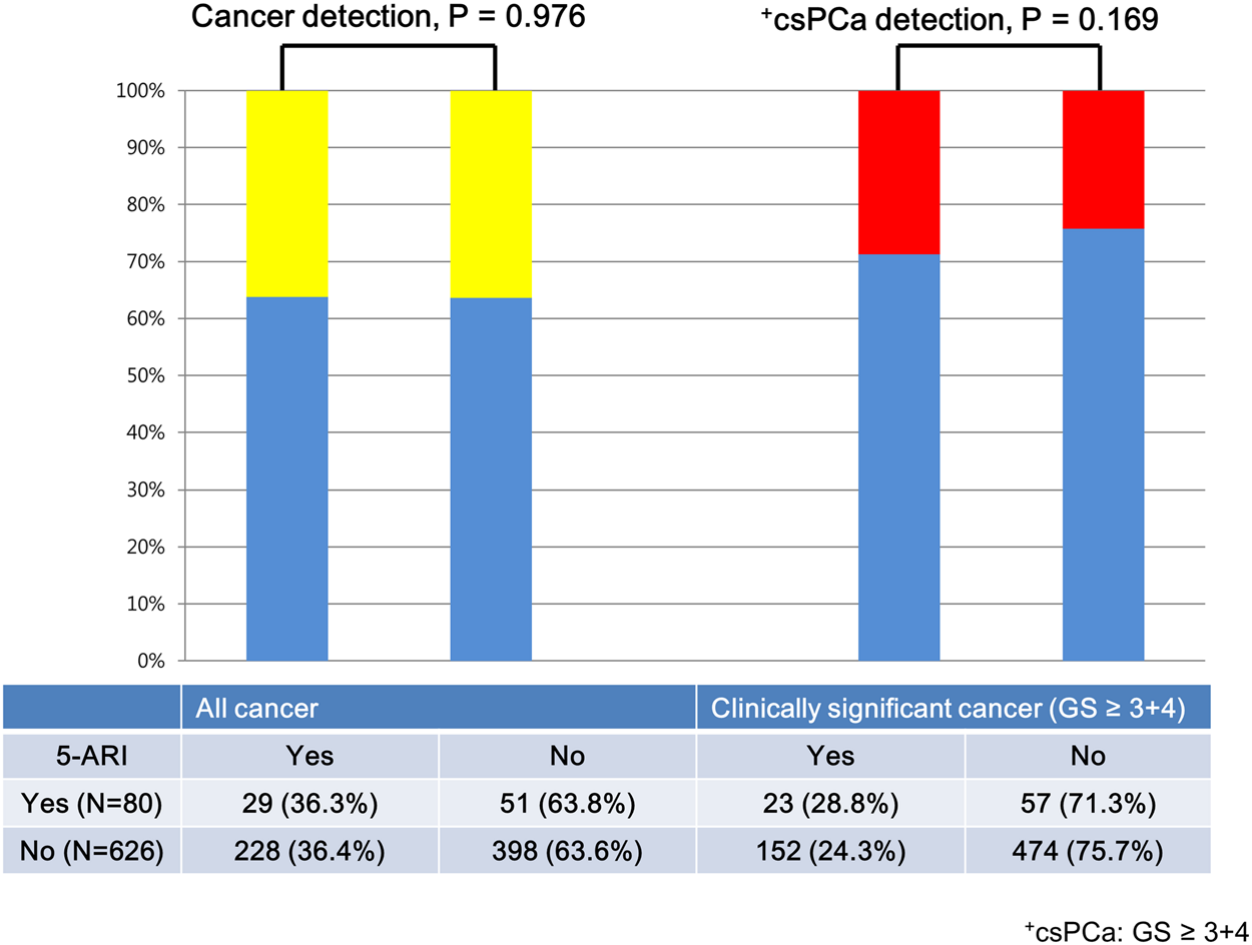
All cases and clinically significant prostate cancer stratified by 5-ARI use in the transrectal ultrasound (TRUS)/magnetic resonance imaging (MRI) fusion biopsy cohort.

**Figure 2:**
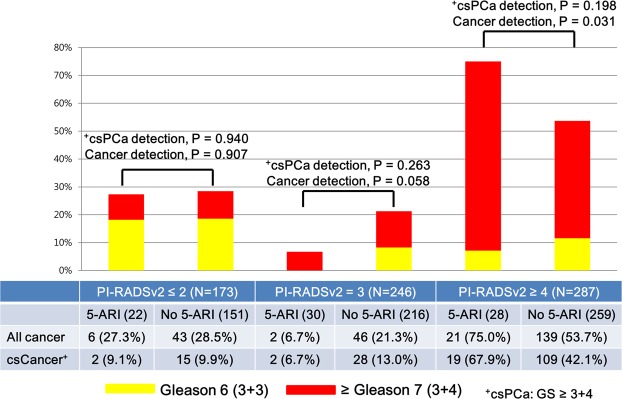
All cases and clinically significant prostate cancer stratified by 5-ARI use according to Prostate Imaging–Reporting and Data System version 2 (PI-RADSv2) category in the transrectal ultrasound (TRUS)/magnetic resonance imaging (MRI) fusion biopsy cohort.

Pathologic outcomes of TRUS/MRI fusion biopsy stratified by 5-ARI use are shown in Table 1, Figs. 1 and 2. There were no significant differences in PCa/csPCa detection rates between 5-ARI and non-5-ARI groups (all, P > 0.05, Table 1 and Fig. 1) in the total cohort. In the subgroup analysis according to the PI-RADSv2 categories, there was significant difference only in the PCa detection rate between the two groups under PI-RADSv2 category ≥4 group (75.0% [5-ARI group] vs. 53.7% [non-5-ARI group], P = 0.031, Fig. 2). By contrast, we found no significant differences in PCa/csPCa detection rates between the two groups in the other PI-RADSv2 categories. In case of patients undergoing long-term (≥3 years) 5-ARI therapy, we also found no significant differences between the two groups in the total or any of PI-RADSv2 categories (all P > 0.05, Supplemental Table 1).

### Logistic regression analyses for the detection of prostate cancer

In multivariate logistic regression analyses for evaluation of variables associated with PCa detection, age (odds ratio [OR], 1.059; 95% confidence interval [CI], 1.035–1.082; P < 0.001), pre-biopsy PSA (OR, 1.041; 95% CI, 1.016–1.066; P = 0.001), prostate volume on TRUS (OR, 0.954; 95% CI, 0.942–0.966, P < 0.001), and PI-RADsV2 category (≥4; OR, 2.506; 95% CI, 1.571–3.995; P < 0.001) were found to be significant predictors (Table 2). However, 5-ARI treatment had no significant association with PCa detection (P = 0.998). In regard of csPCa detection, age (odds ratio [OR], 1.062; 95% confidence interval [CI], 1.035–1.090; P < 0.001), pre-biopsy PSA (OR, 1.062; 95% CI, 1.034–1.090; P < 0.001), prostate volume on TRUS (OR, 0.956; 95% CI, 0.943–0.970, P < 0.001), and PI-RADsV2 category (≥4; OR, 5.528; 95% CI, 3.017–10.130; P < 0.001) were found to be significant predictors (Table 3). However, 5-ARI treatment also had no significant association with csPCa detection (P = 0.384).

**Table 2:** Univariate and multivariate logistic regression analyses of factors for prostate cancer detection in total cohort.

Variables	Univariate			Multivariate		
	OR	95% CI	P	OR	95% CI	P
Age	1.055	1.036–1.075	<0.001	1.059	1.035–1.082	<0.001
Pre-biopsy PSA	1.044	1.023–1.065	<0.001	1.041	1.016–1.066	0.001
Prostate volume, total	0.964	0.953–0.975	<0.001	0.954	0.942–0.966	<0.001
PI-RADsV2 category						
≤2	Reference			Reference		
3	0.598	0.378–0.946	0.028	0.591	0.357–0.978	0.041
≥4	3.188	2.127–4.778	<0.001	2.506	1.571–3.995	<0.001
5-ARI	0.999	0.616–1.622	0.998			

5-ARI, 5 alpha-reductase inhibitor; PI-RADSv2, Prostate Imaging–Reporting and Data System version 2; PSA, prostate-specific antigen.

**Table 3:** Univariate and multivariate logistic regression analyses of factors for clinically significant prostate cancer detection in total cohort.

Variables	Univariate			Multivariate		
	OR	95% CI	P	OR	95% CI	P
Age	1.067	1.044–1.090	<0.001	1.062	1.035–1.090	<0.001
Pre-biopsy PSA	1.070	1.046–1.094	<0.001	1.062	1.034–1.090	<0.001
Prostate volume, total	0.968	0.956–0.980	<0.001	0.956	0.943–0.970	<0.001
PI-RADsV2 category						
≤2	Reference			Reference		
3	1.275	0.679–2.392	0.450	1.315	0.666–2.593	0.430
≥4	7.387	4.253–12.830	<0.001	5.528	3.017–10.130	<0.001
5-ARI	1.258	0.750–2.111	0.384			

5-ARI, 5 alpha-reductase inhibitor; PI-RADSv2, Prostate Imaging–Reporting and Data System version 2; PSA, prostate-specific antigen.

In subgroup analysis based on the PI-RADSv2 categories, we also found no significant association of 5-ARI with PCa detection (Supplemental Tables 2 and 3).

### RP pathologic outcomes in subgroup analysis

The results of comparative analyses involving pathologic outcomes between 5-ARI and non-5-ARI groups are summarized in Table 4. We found no significant differences in biopsy GS between the 5-ARI and non-5-ARI groups in a total of 257 patients who were diagnosed with PCa (P = 0.106). In addition, among a total of 147 patients who underwent subsequent RP after TRUS/MRI fusion biopsy in our institution, there were no significant differences between the two groups in terms of RP GS (P = 0.489) and GS upgrading (P = 0.608).

**Table 4:** Comparative analysis of pathological outcomes between 5-ARI and non-5-ARI groups in the TRUS/MRI fusion biopsy cohort and the subgroup of radical prostatectomy cohort.

N (%)	5-ARI group	Non-5-ARI group	P
Biopsy Gleason score	N = 29	N = 228	0.106
6 = 3 + 3	6 (20.7%)	76 (33.3%)	
7 = 3 + 4	6 (20.7%)	72 (31.6%)	
7 = 4 + 3	7 (24.1%)	31 (13.6%)	
≥8	10 (34.5%)	49 (21.5%)	
Radical prostatectomy Gleason score	N = 19	N = 128	0.489
6 = 3 + 3	0 (0)	4 (3.1%)	
7 = 3 + 4	7 (36.8%)	66 (51.6%)	
7 = 4 + 3	9 (47.4%)	44 (34.4%)	
≥8	3 (15.8%)	14 (10.9%)	
Gleason score upgrading			0.608
No (N = 95)	11 (57.9%)	84 (65.6%)	
Yes (N = 52)	8 (42.1%)	44 (34.4%)	

5-ARI, 5 alpha-reductase inhibitor.

## Discussion

In the past decade, a growing body of evidence suggested the role of mpMRI in the detection and management of PCa^3^. The PI-RADSv2 criteria, released in 2016 by the European Society of Urogenital Radiology (ESUR), have ensured a standardized and reliable radiological evaluation^16,18^. However, due to obvious concerns, this guideline is still disputed. The PROMIS data results missed 11% of GS ≥ 4 + 3 and 28% of GS ≥ 3 + 4 on mpMRI^1^. In the PRECISION trial, a substantial number of patients (28%) in the MRI-targeted biopsy group did not undergo biopsy because of negative results on mpMRI^2^. Accordingly, a significant number of csPCa cases might have been missed. In the future, additional tools for risk assessment need to be combined in order to improve the performance of mpMRI^19–21^.

5-ARIs, including finasteride and dutasteride, are known to affect the cellular involution and epithelial shrinkage of benign prostatic tissue, and increase the stromal/epithelial ratio in PCa^22^. It is suggested that 5-ARIs may induce significant phenotypic alterations in both BPH and PCa. In this regard, the use of 5‐ARIs is expected to affect the interpretation of mpMRI in 5‐ARI‐treated patients^11–13^. In a recent randomized clinical trial of 37 patients (dutasteride arm [n = 18] vs. placebo arm [n = 19]), Gianti *et al*.^12^ reported that absolute changes in ADC and conspicuity varied significantly between the two groups at 6 months: (−0.03 vs. 0.08, P = 0.033) and (0.11 vs. −0.16, P = 0.012). Consequently, they recommended a lower threshold for biopsy indication in patients exposed to dutasteride. Conversely, in a subsequent study, Giganti *et al*.^11^ evaluated the role of quantitative T2WI from patients recruited for the MAAPED trial. Accordingly, they found no significant differences in T2WI at baseline and after 6 months both in the placebo and the dutasteride arm. However, in the current practice of PI-RADSv2 scoring, mpMRI includes both DWI/ADC with/without DCE for the evaluation of peripheral zone (PZ) and T2WI with/without DWI for the evaluation of transition zone^16^. In the current study, all lesions were scored using both ADC maps and T2WI based on PI-RADSv2. We found no significant differences in PCa/csPCa detection rate between the 5-ARI and non-5-ARI groups (all, P > 0.05, Table 1, Figs. 1 and 2), even in the long-term (≥3 years) 5-ARI subgroup (all, P > 0.05, Supplemental Table 1). In addition, 5-ARI had no significant association with PCa/csPCa detection even in univariate logistic regression analyses (all, P > 0.05, Tables 2 and 3). These results reflect real-world clinical practice settings.

Previous studies have also investigated the clinical use of 5-ARIs to chemoprevention^6–9,23^. Two landmark studies, the PCPT (Prostate Cancer Prevention Trial)^23^ and the REDUCE trial^6^, demonstrated a significant chemopreventive effect of 5-ARIs on the reduction of cumulative PCa incidence. However, an increased incidence of high-grade PCa was observed in both trials, and the mechanism of 5-ARI on PCa prevalence reduction is still elusive. In the current study, we found no significant differences in biopsy GS and RP pathologic GS/GS upgrading (all, P > 0.05, Table 4). Further large-scale studies are warranted to validate and generalize our results.

The current study has some limitations. First, even with a study based on a large tertiary institution, the retrospective design was a crucial drawback. In addition, we did not conduct a re-review of pathology slides. Accordingly, subsequent misclassification of a few lesions might have affected outcomes. Finally, we could not investigate the serial changes in imaging after treatment with 5-ARIs due to the lack of baseline mpMRI data.

## Conclusions

Treatment with 5-ARI did not adversely affect the rates of PCa and csPCa detection after TRUS/MRI fusion biopsy, even with long-term use, suggesting that 5-ARI may not impair the performance of mpMRI. In addition, 5-ARI exposure had no effect on the pathological outcomes at both biopsy and RP.

## Supplementary information

Supplemental table 1–3
