# Supplementary material for: The effect of 5 alpha-reductase inhibitor therapy on prostate cancer detection in the era of multi-parametric magnetic resonance imaging
Source: Sci Rep. 2019 Nov 28;9:17862. doi: 10.1038/s41598-019-54464-9 (PMC6882845; doi:10.1038/s41598-019-54464-9)
Supplement: Supplementary file 1 — Supplemental table 1–3 [file 41598_2019_54464_MOESM1_ESM.pdf]

TITLE

The effect of 5 alpha-reductase inhibitor therapy on prostate cancer detection in the era of multi-parametric magnetic resonance imaging

AUTHORS AND INSTITUTIONS

Jung Kwon Kim[1], Hak Jong Lee[2], Sung Il Hwang[2], Gheeyoung Choe[3], Sung Kyu Hong[1,4]*

[1]Department of Urology, Seoul National University Bundang Hospital, Seongnam, Korea

[2]Department of Radiology, Seoul National University Bundang Hospital, Seongnam, Korea

[3]Department of Pathology, Seoul National University Bundang Hospital, Seongnam, Korea

[4]Department of Urology, Seoul National University College of Medicine, Seoul, Korea

CORRESPONDENCE*

Sung Kyu Hong, MD, PhD.

Professor; Department of Urology, Seoul National University College of Medicine, Seoul, Korea; Seoul National University Bundang Hospital, Seongnam, Korea 173-82, Gumi-Ro, Bundang-gu, Seongnam-si, Gyeonggi-do, 13620, Korea

Tel: 82-31-787-7343, Fax: 82-31-787-4057, E-mail: skhong@snubh.org

Supplemental Table 1. Comparative analysis of prostate cancer detection rates between 5-ARI and No 5-ARI groups according to PI-RADSv2 category in patients taking 5-ARI more than 3 years

	5-ARI group	No 5-ARI group	P
Total cohort	N=35	N=626	0.199
Cancer	9 (25.7%)	228 (36.4%)	
No cancer	26 (74.3%)	398 (63.6%)	
PI-RADS ≤ 2	N=13	N=151	0.310
Cancer	2 (15.4%)	43 (28.5%)	
No cancer	11 (84.6%)	108 (71.5%)	
PI-RADS = 3	N=10	N = 216	0.102
Cancer	0 (0)	46 (21.3%)	
No cancer	10 (100)	170 (78.7%)	
PI-RADS ≥ 4	N=12	N = 259	0.751
Cancer	7 (58.3%)	139 (53.7%)	
No cancer	5 (41.7%)	120 (46.3%)	

Supplemental Table 2. Univariate and multivariate logistic regression analyses to detect prostate cancer in patients of PI-RADSv2 category ≥ 4 groups

	**Univariate**			**Multivariate**		
Variables	OR	95% CI	P	OR	95% CI	P
Age	1.051	1.023-1.081	<0.001	1.072	1.034-1.111	<0.001
BMI	0.944	0.852-1.047	0.277			
Pre-biopsy PSA	1.037	1.010-1.066	0.008	1.064	1.019-1.111	0.005
Prostate volume, total	0.950	0.934-0.966	<0.001	1.006	0.971-1.042	0.745
Prostate volume, TZ	0.909	0.882-0.937	<0.001	0.897	0.868-0.927	<0.001
BPH medication						
a-blocker	0.505	0.315-0.811	0.005	0.819	0.434-1.545	0.538
anticholinergics	0.992	0.380-2.591	0.986			
5-ARI	2.590	1.064-6.304	0.036	2.132	0.675-6.732	0.197

Supplemental Table 3. Univariate and multivariate logistic regression analyses to detect prostate cancer in patients of PI-RADSv2 category 3 groups

	**Univariate**			**Multivariate**		
Variables	OR	95% CI	P	OR	95% CI	P
Age	1.028	0.989-1.068	0.158	1.116	1.043-1.195	0.002
BMI	1.212	1.032-1.424	0.019	1.354	1.087-1.686	0.007
Pre-biopsy PSA	1.023	0.982-1.066	0.276			
Prostate volume, total	0.968	0.946-0.991	0.006	1.008	0.954-1.065	0.774
Prostate volume, TZ	0.935	0.900-0.971	<0.001	0.901	0.854-0.950	<0.001
BPH medication						
a-blocker	0.580	0.305-1.103	0.097			
anticholinergics	2.159	0.622-7.492	0.225			
5-ARI	0.264	0.061-1.149	0.076			
